# Phonological encoding in Vietnamese: An experimental investigation

**DOI:** 10.1177/17470218211053244

**Published:** 2021-10-21

**Authors:** Rinus G Verdonschot, Hoàng Thị Lan Phương, Katsuo Tamaoka

**Affiliations:** 1 Max Planck Institute for Psycholinguistics, Nijmegen, The Netherlands; 2 Graduate School of Humanities, Nagoya University, Nagoya, Japan

**Keywords:** Language production, Vietnamese, phonological Stroop, priming, prosodification, phonological encoding

## Abstract

In English, Dutch, and other Germanic languages the initial phonological unit used in word production has been shown to be the phoneme; conversely, others have revealed that in Chinese this is the atonal syllable and in Japanese the mora. The current paper is, to our knowledge, the first to report chronometric data on Vietnamese phonological encoding. Vietnamese, a tonal language, is of interest as, despite its Austroasiatic roots, it has clear similarities with Chinese through extended contact over a prolonged period. Four experiments (i.e., masked priming, phonological Stroop, picture naming with written distractors, picture naming with auditory distractors) have been conducted to investigate Vietnamese phonological encoding. Results show that in all four experiments both onset effects as well as whole syllable effects emerge. This indicates that the fundamental phonological encoding unit during Vietnamese language production is the phoneme despite its apparent similarities to Chinese. This result might have emerged due to tone assignment being a qualitatively different process in Vietnamese compared to Chinese.

Speaking is one of the most remarkable abilities humans perform daily. Seemingly effortlessly we go from the selection of concepts in our head to moving a large variety of articulators in rapid succession to produce speech. This ability has generated a lot of interest among psycholinguists and experimental psychologists and several theoretical models, supported by experimental and other evidence, have emerged to describe how we accomplish this feat. Most models agree that there is a broad distinction between accessing the meaning of a word and assembling its pronunciation (e.g., Caramazza, 1997; Dell, 1986; Levelt et al., 1999; Roelofs, 2015).

One of the most detailed theoretical accounts of word production can be found in Levelt et al. (1999). In their model after a concept has being selected, and its semantic and syntactic properties have been selected, phonological encoding commences. It is this process that has generated quite some interest in recent years and there is now accumulating evidence that the size of the initial phonological unit selected during this stage is different between languages. The original Levelt et al. (1999) model has been mainly based on Germanic languages (such as Dutch, German, and English) and suggests that two pieces of information are initially necessary. Particularly, spelled out segments of a word and a word’s metrical information need to be retrieved. For example, to say the word “believe” one must retrieve (from memory) its phonemic segments, e.g., /b/, /ɪ/, /l/, /i/, /v/ and its metrical structure (i.e., disyllabic with stress on the second syllable). These pieces of information will then be combined to form the word /bɪˈliv/. One reason that the latter (prosodified) pattern cannot simply be stored, at least in Germanic languages, is that in these languages words often re-syllabify (e.g., the /v/ in “believe it” would attach itself to the next syllable: /bɪˈli/ + /vɪt/).

Different paradigms have been devised to investigate how phonological encoding takes place. For example, the implicit priming paradigm (Meyer, 1990, 1991) in which a person needs to first remember several prompt-response pairs (e.g., dog—bark) and then after being shown a prompt (e.g., dog) needs to respond with the learned response (e.g., bark). The crucial manipulation in this paradigm is the creation of groups which are either overlapping in a certain feature (e.g., same onset: bark, bill, boot) or not (e.g., bark, dull, main). It has been shown that initial overlap is necessary to facilitate naming latencies and a single phonemic onset (e.g., /b/) is sufficient. However, this seems to be different in Chinese (e.g., Chen et al., 2002; O’Seaghdha et al., 2010) and Japanese (Kureta et al., 2006). In those languages, the pattern is also incremental but a minimal overlap of an atonal syllable in Chinese (e.g., /fei/ in /fei1dan4/ “missile,” /fei2zao4/ “soap,” /fei4bu4/ “lung”) or a mora in Japanese (e.g., /ka/ in /ka.ts.ura/ “wig,” /ka.fu.n/ “pollen,” /ka.bu.ki/ “Japanese theatre”) are necessary for facilitation effects to appear. Similar results have been obtained with the masked priming word naming paradigm in which a prime is quickly (e.g., 50 ms) presented before a to-be-named target word. For example, if the word “pear” is preceded by a prime word such as “pole” it will be pronounced quicker than if it is preceded by the prime “take” (e.g., Forster & Davis, 1991). As with the implicit priming paradigm, in Chinese (e.g., You et al., 2012) and Japanese (e.g., Ida et al., 2015; Verdonschot et al., 2011) again the syllable and mora, respectively, are found to be the relevant unit to put into a tonal/pitch frame (see Roelofs, 2015) and not a phonemic segment.

For other languages, such as Cantonese Chinese, the picture seems to be more complicated. For example, Wong and Chen (2008) using a picture-word interference paradigm obtained significant facilitation effects for the syllable overlap condition (Example picture: 箭 /zin3/ “arrow” with distractor: 氈 /zin1/ “felt”) but also found significant facilitation effects for shared rhyme (淺 /cin2/ “shallow”). Effects were absent when only the onset was shared (滯 /zai6/ “stagnation”). This suggests that sub-syllabic units might be involved in the initial phonological planning in Cantonese. The idea that sub-syllabic units are the appropriate planning units in Cantonese was further confirmed through use of the implicit priming task (i.e., Wong et al., 2012) and seems to be a robust finding (though see Wong et al., 2018 who suggested that syllable retrieval still may come before sub-syllabic encoding even in Cantonese spoken word production).

One issue which has been put forward with the experiments mentioned earlier is the use of script. Some have suggested that if a certain script type is used in an experiment (e.g., Japanese kana or Chinese hanzi) this might influence the results as different script types may activate different underlying units. That is, in alphabetic script each letter (or letter cluster) maps onto a phoneme, but Japanese kana (e.g., すし /su.shi/ “sushi”) reflect mora; and Chinese hanzi (e.g., 菜单 /cai4dan1/ “menu”) maps onto morpho-syllables. However, there is currently mixed evidence on the role of script in this matter. For example, Wong and Chen (2008) found sub-syllabic effects using Chinese (syllabic) distractor words and Verdonschot et al. (2011) used Romanized Japanese (i.e., alphabetic) to investigate whether script type could play a role, only found mora effects; therefore, both did not find a significant role of script. However, recently, Yoshihara et al. (2017) found, using masked priming, that only when the whole pronunciation of a prime and target were overlapping (e.g., 迫害 /ha.ku-ga.i/ - 博物 /ha.ku-bu.tsu/) significant facilitation effects occurred (i.e., so a prime as 灰皿 /ha.i-za.ra/ would be ineffective as it does not encompass the whole first syllable /ha.ku/). If stimuli were transcribed into kana (e.g., はくがい /ha.ku-ga.i/), then moraic effects appeared again. This “whole kanji” effect might, however, not apply to all tasks as Verdonschot and Kinoshita (2018) pointed out. They argued that when the task “does not necessarily involve reading” (like in Yoshihara et al., 2017) it might not exert an influence and they consequently used a Stroop colour naming task in which both moraic hiragana (e.g., みせ /mi.se/) and morphemic kanji (e.g., 店 /mi.se/ “shop”) overlapped with a to-be-named colour (e.g., /midori/ “green”) and facilitatory mora effects appeared both for katakana and kanji words. However, Yoshihara et al. (2021) recently showed that using romaji (i.e., Romanized Japanese) naming responses were also faster when the initial phoneme was shared between the colour name and the distractor (e.g., the “k” in “kasa” [umbrella] significantly speeded up naming yellow “kiiro” in Japanese) indicating an effect of phonological grain size a particular distractor maps onto. One thing which the results from Verdonschot and Kinoshita (2018) and Yoshihara et al. (2017, 2021) make clear is that it is worthwhile to investigate language/script combinations in which the script does not reflect the size of the underlying fundamental phonological unit (i.e., such as Japanese kanji which reflect morpho-syllables instead of moras). However, such combinations are extremely rare as typically the script which coexists with a language naturally reflects the size of the fundamental underlying phonological unit.

In this paper we would like to draw attention to the *Vietnamese* language. Vietnamese is a syllabic tonal language which ostensibly has many commonalities with Chinese (further explained below) but only comparatively recently has started using the Latin (alphabetic) script. Vietnamese is the official language of Vietnam. It uses tones to distinguish between lexical items and it has a relatively simple syllabic structure which resembles Chinese. For example, like Chinese, Vietnamese syllables are phono-tactically constrained. They consist of an (optional) onset consonant, followed (optionally) by a bilabial consonant glide, followed by an obligatory vowel (carrying one of six tones), followed optionally by a single coda consonant (cf. Pham & Baayen (2015), p. 1077; also see Pham & Baayen, 2015 for all list of all possible Vietnamese syllable types). Vietnamese syllables are also all single morphemes, and all morphemes are monosyllabic (cf. Pham & Baayen, 2015, p. 3). Words are not inflected for person, number, case, tense, gender, etc. and novel words can only be formed using compounding or reduplication. Vietnamese has been heavily influenced by Chinese during a thousand years in which Vietnam belonged to the Chinese empire. Its vocabulary therefore has many borrowings from Chinese through colonisation as well as prolonged cultural and economic interactions. McWhorter (2000) estimates that as much as 33% of the entire Vietnamese vocabulary consists of Chinese words. Vietnamese has been proposed to be a syllable-timed language (e.g., Mixdorff & Ingram, 2009; Nguyễn et al., 2008) in which each syllable has a specification for a lexical tone. Note that a language that has syllable timing does not have to constitute a phonological unit of that size per se. For example, French is syllable-timed but significant onset effects have been found in this language (Carreiras et al., 2006; Grainger & Ferrand, 1996). However, though many syllable-timed languages display variable degrees of durational variability, in Vietnamese, systematic variances for vowel quality or the duration among syllables are relatively uncommon (Nguyễn & Ingram, 2005). This might be the result from the prolonged presence and influence of China (and the Chinese language) in Vietnam. In addition, though simply having syllabic timing may not be enough, according to Cholin et al. (2004) the main argument why phonological encoding needs a segment-to-frame association process is that, during speech, syllable boundaries are different from the stored representations in the lexicon. In Dutch, for example, re-syllabification is estimated to occur in around 17% of all words in connected speech (i.e., Schiller et al., 1996). Importantly, for Chinese, Cholin et al. (2004) put forward that, as Mandarin Chinese does not have re-syllabification, syllabic units may be represented earlier in the phonological encoding process (p. 59). This fits with Chen et al.’s (2002) finding that atonal syllables are the sole units which showed significant priming as further down the road (i.e., after phonological encoding finishes) the tone would always have to be specified. It is probable that as Vietnamese is a tonal syllabic-timed language (with no re-syllabification) it will behave similarly to Mandarin Chinese with respect to the prosodification process (e.g., the syllable and not the phoneme would be used as the fundamental phonological unit to insert in a tonal frame). The similarities between Vietnamese and Chinese are also discussed in Sidwell (2008) who points out that Vietnamese (though it is officially an Austroasiatic language) has completely lost the rich morphological system proto-Austroasiatic (also called proto Mon–Khmer) languages had. To illustrate this claim, Sidwell (2008) mentions the example of compounding which has replaced affixation over time as a new word forming mechanism in Vietnamese due to both the fact that syllables became increasingly simple over time (through Vietnam’s extended contact with Chinese, a compounding language). In this respect, morphological encoding in modern Vietnamese is very similar to that of Chinese and deviates significantly from its proto-Austroasiatic roots.

Official documents in Vietnam were written in classical Chinese until roughly early 20th century while the more popular literature was written using the “chữ nôm” script, which are Chinese characters especially adapted to suit the Vietnamese vocabulary. That is, each Vietnamese word could be written using one character per syllable, some of which originated from China, but also many characters were invented in Vietnam. The main point here is that all these scripts were morpho-syllabic (like Chinese) with each character corresponding to a single syllable (e.g., 越南 Việt Nam “Vietnam”) which corresponded well with the syllabic Vietnamese language. However, through the influence of French colonisation the Latin alphabet rather abruptly and mandatorily replaced the older scripts in official documents and popular literature. Consequently, from the beginning of the 20th century (until now) modern Vietnamese is written using “chữ quốc ngữ” (lit: National Language Script; originally invented by Portuguese missionaries in the 17th century) which is an alphabetic (latin) script using diacritics to denote the tones (e.g., tháng “month”) as well as to distinguish certain letters which have slightly different sounds (e.g., vườn “garden”). The new Vietnamese orthography is transparent, having a strong grapheme-to-phoneme match, though syllables of compound words are still separated by a space (e.g., the word xe lửa “train” is not written as xelửa) effectively creating syllable blocks throughout Vietnamese text.

Though linguists have issued theoretical accounts for various aspects of the Vietnamese language there is an almost complete lack of chronometric data. That is, contrasting Indo-European, Chinese, and Japanese languages, few studies have used an experimental approach to measure naming latencies and/or accuracy on Vietnamese language production. The question is whether the initial phonological encoding unit in Vietnamese is the syllable (i.e., like Chinese as they are both syllabic-tonal languages and both without re-syllabification and inflections) or whether another unit, such as the phoneme, is initially selected (perhaps influenced by introduction of an alphabetic script; see Kureta et al., 2015). The notion that phonemes might be selected during the initial phase in Vietnamese phonological encoding (despite its similarities to Chinese) can be seen in Vietnamese language games like *nói lái* (or “speaking backwards”) in which tones can be switched between word pairs, but, interestingly, also between the initial consonant and rime (see Macken & Nguyễn, 2006). For example, cá đối “grey mullet” may become cối đá “stone mortar” in this game. This contrasts with comparable games played in Chinese (文字接龙) and Japanese (*shiritori*) in which phonemic constituents can never be switched and either syllables or moras need to be exchanged.

As it is unclear whether Vietnamese uses the phoneme, mora, or syllable as the initial fundamental unit of language production the current paper aims to investigate Vietnamese language production by employing a variety of frequently used paradigms. Specifically, a masked priming paradigm (Experiment 1) manipulating overlap status (C, CV and Full) between masked word prime and target, a Phonological Stroop task (Experiment 2) using the same manipulation between colour name and non-word distractor and two picture-word naming tasks (Experiment 3 and 4) using the same manipulation (with written and auditory distractors respectively). If Vietnamese shows sub-syllabic or phonemic onset priming in all experiments, it is likely that the initial unit in the phonological encoding process is the phoneme (like Germanic languages). However, if only syllabic effects are found then Vietnamese phonological encoding would be like Mandarin Chinese. If there a difference is observed between experiments more involved in reading (e.g., Experiment 1) or not then script may play a role.

## Experiment 1—word priming task using Vietnamese

### Participants

Thirty students, native speakers of Vietnamese, from Ho Chi Min University of Pedagogy (age: 21 ± 2; Female: 22, Male: 8) took part in this experiment for monetary compensation. All had normal or corrected-to-normal vision. Participants took the LexTale test (Lemhöfer & Broersma, 2012; range: 0–100) and scored on average: 46 ± 7.1 indicating very low proficiency in English. Their self-assessment values (range: 0–100; *reading*: 46 ± 8.1; *writing*: 43 ± 7.7; *listening*: 43 ± 6.7; speaking: 44 ± 5.4) reflected similar low-proficiency and experience in using English as an L2. No abilities in other languages were reported.

### Materials and design

Twenty-four to-be-named target words were selected (e.g., BẠN “friend”) with six conditions, C-Overlap (e.g., beo “leopard”), C-Control (e.g., nấm “mushroom”), CV-Overlap (e.g., bát “bowl”), CV-Control (e.g., nốt “note”), Full-Overlap (e.g., bàn “table”), Full-Control (e.g., nôi, “baby crib”). The tone did not overlap between target and distractor in the overlap conditions (and most of the control conditions); therefore, the full-overlap condition involved the *atonal syllable* with the target (see Chen et al., 2002; O’Seaghdha et al., 2010 who showed that the atonal syllable was the relevant proximate unit in Chinese). Another advantage is that there was no visual overlap of the Vietnamese tone diacritics between target and distractors in our experiment. Due to the lack of reliable corpus information in Vietnamese (e.g., frequency, familiarity, neighbourhood size) we decided that instead of one control condition each overlap condition would be reshuffled to form its own control. In this way any differences between Overlap and Control would not be due to inadequate matching characteristics. Online Supplementary Material A displays an overview of all stimuli used in this experiment. The experiment was divided into 6 blocks of 24 items, in total 144 items were obtained per participant. Block order was randomised via a Latin square design and stimuli within blocks were randomised per participant.

### Apparatus and procedure

E-Prime 2.0 was used to administer the experiment. First, a fixation (+) was shown for 750 ms, which was then replaced by a forward mask (###) for 500 ms. Subsequently, the prime was shown for 50 ms after which the target appeared and disappeared when it was named (or after maximally 2,500 ms). After this the experimenter judged whether the trial was correctly named or whether a voice key error (e.g., couching, mis-triggering) or genuine error (e.g., wrong word) had occurred before the next trial started.

### Results^1^

About 2.0% of the remaining RTs were discarded due to (1) a failure to respond, (2) stuttering or repairing a response, (3) triggering the voice key using a non-verbal response (e.g., coughing), and (4) or a failure to trigger the voice key. In addition, there were 0.6% errors (e.g., naming a wrong word) across the board.

The treatment of correct RT data for this analysis was as follows. First, we excluded trials (3.4%), which were quicker than 300 or slower than 1,500 ms. This left 4060 data points for further analysis, see Table 1 for RTs and accuracy information. A comparison of raw RTs, log-transformed RTs, and inverse-transformed RTs (i.e., -1,000/RT) revealed that log-transformed RTs were closest to normality and were therefore used in subsequent analyses. Error data was not further analysed as there were few errors which were distributed equally among conditions.

**Table 1.** RTs and accuracy information for Experiment 1.

Target (e.g., NỒI ‘pot’)	RT (*SD*)	E (in %)	Effect
C-overlap (e.g., nấm—‘mushroom’)	427 (59)	0.3	16
C-control (e.g., beo—‘leopard’)	443 (60)	0.7	
CV-Overlap (e.g., nón—‘hat’)	425 (58)	0.3	13
CV-Control (e.g., mục—‘catalogue’)	438 (59)	0.6	
Full-Overlap (e.g., nôi—‘crib’)	418 (58)	1.0	19
Full-Control (e.g., bàn—‘table’)	437 (55)	0.7	

*SD*: standard deviation, RT: reaction times, E: error.

Response latencies were analysed with a linear mixed effects model with participants and items as crossed random effects (e.g., Baayen, 2008) using the “lme4” package (Bates et al., 2013) implemented in R 3.0.3 (R Development Core Team, 2014) to analyse RT for correct trials and error rates. The “lmerTest” package in R was used to calculate the p-values using Satterthwaite’s approximation for the degrees of freedom (Kuznetsova et al., 2014).

In all experiments we opted to use an incremental modelling approach (Matuschek et al., 2017) to establish the most optimal statistical model for our data. We considered the following variables: “Trial” which denotes how far a participant had progressed in the experiment, “Congruency” with two levels (i.e., overlap, control) and “OverlapSize” with three levels (i.e., Onset, CV, and Full). The factor Congruency was deviation-contrast-coded (-.5, .5). The final model was log (RT) ~ Trial + Congruency * OverlapSize + (1 + OverlapSize|Participant) + (1|Item). See Table 2 for more details. As the global interaction between congruency (overlap or not) and size of overlap (C, CV, Full) was not significant, pairwise comparisons are not warranted.

**Table 2.** Coefficients of the main effects of the final model, together with the standard error, t-values and p-values (Experiment 1).

	Estimate	*SE*	*t*-value	*p*-value
Intercept	6.064074	0.015248	397.695	<.001
Trial (centred)	−0.008303	0.001683	−4.932	<.001
Congruency (Overlap)	−0.034068	0.006163	−5.528	<.001
OverlapSize (CV)	−0.006958	0.004114	−1.691	0.0908
OverlapSize (FULL)	−0.017560	0.004128	−4.254	<.001
Congruency: OverlapSize (CV)	0.002758	0.008227	0.335	0.7375
Congruency: OverlapSize (FULL)	−0.011033	0.008255	−1.337	0.1814

*SE*: standard error.

### Discussion

We found equal priming for C, CV, and Full overlap conditions (evidenced by the absence of an interaction between Congruency and OverlapSize). Therefore, it seems that, like Germanic languages, Vietnamese uses the phoneme as the initial unit in the prosodification process. However, we presented the primes and targets in chữ quốc ngữ (standard Vietnamese script). Therefore, both masked primes and targets in this experiment were *alphabetic* and the task (i.e., reading aloud) might have made the experiment’s focus more on written word naming rather than about phonological encoding. Note that it may also seem puzzling why full overlap priming would show identical RT benefits compared to onset priming, however, these combinations do *not* constitute identity primes. That is, *prime*: nôi—‘crib’—*target*: NỒI “pot” are not identical words as they do not carry the same tone (and therefore meaning), which is an important factor in Vietnamese. Furthermore, the absence of the interaction might be because masked primes may not have been fully processed. For example, only the initial letter might have been processed without full prime processing, and it has been shown in English that additional overlap does not necessarily creates benefits during priming (e.g., Kinoshita, 2000; Nakayama et al., 2016).

Therefore, to further investigate phonological encoding in Vietnamese we also employed a Phonological Stroop paradigm. It has been argued earlier (Verdonschot & Kinoshita, 2018; but see Yoshihara et al., 2021 for a different view) that there are two (not mutually contradictory) possibilities. The first is that, through prolonged experience, the script’s grain size, which is used to write the language (i.e., whether each character maps onto a phoneme, mora, or syllable), affects the phonological unit employed by the language user. The second is whether specific spoken word-production tasks are sensitive to certain orthographic influences. In other words, the first issue refers to the language-user’s phonological representation itself and the latter relates to the mechanism of the task which produces the overlap benefit. Verdonschot and Kinoshita (2018) administered a task that did not require reading aloud a written word stimulus but rather naming of a colour (or picture name) which was deemed to be more suitable to investigate phonological encoding (as the pronunciation of a colour, or picture, name would not have to be assembled in a step-by-step fashion, as compared to a reading aloud task).

If initial phonological encoding in Vietnamese is indeed phoneme based, then we expect a similar pattern of results as in Experiment 1 (though there might be increased benefits for CV and Full overlap due to task differences). If somehow the mechanism of the task in Experiment 1 (word naming) produces the overlap benefit for the phoneme and the phonological encoding process would, in fact, be syllabic (or CV) for Vietnamese then we expect significant effects for the Full Overlap (or CV) conditions but not for the onset overlap condition in Experiment 2.

## Experiment 2—phonological Stroop task using Vietnamese

### Method

#### Participants

Same participants as in Experiment 1.

#### Materials and design

Five colours (hồng “pink,” tím “purple,” đen “black,” ‘xanh’ blue and trắng “white”) were selected. Each colour (e.g., hồng) could be printed in a C-congruent non-word (e.g., hếp) or its control (e.g., gếp) a CV-congruent non-word (e.g., hỗt) or its control (e.g., gỗt) or a full-overlap (atonal syllable) non-word (e.g., hống) or its control (e.g., pống). The tones were different between the target colour name and the non-words (but similar between overlap and controls). Colour names were repeated for 5 times with different distractors yielding 150 experimental items per person. Stimulus order per block was pseudo-randomised using Mix (van Casteren & Davis, 2006) with two constraints: (a) two colours could never follow each other and (b) two identical conditions could never follow each other. Block order was distributed using a latin-square design among participants.

#### Apparatus and procedure

E-Prime 2.0 was used to administer the experiment. First, a fixation (+) was shown for 750 ms. Subsequently, the target (a non-word printed in any of the five colours on a grey background) appeared and disappeared when it was named (or after maximally 2,500 ms). After this the experimenter judged whether the trial was correctly named or whether a voicekey error (e.g., couching, mistriggering) or genuine error (e.g., wrong word) had occurred before the next trial started.

#### Results

About 3.2% of the RTs were discarded due to (1) a failure to respond, (2) stuttering or repairing a response, (3) triggering the voice key using a non-verbal response (e.g., coughing), and (4) or a failure to trigger the voice key. In addition, there were very few errors (0.7%; e.g., naming a wrong colour or the non-word) across the board which were equally distributed among conditions and therefore not analysed.

The treatment of correct RT data for this analysis was as follows. First, we excluded 55 trials (1.2%), which were faster than 300 or slower than 1,500 ms. This left 4,269 data points for further analysis, see Table 3 for RTs and accuracy information. A comparison of raw RTs, log-transformed RTs, and inverse-transformed RTs (i.e., -1,000/RT) revealed that inverse-transformed RTs were closest to normality and were therefore used in subsequent analyses.

**Table 3.** RTs and accuracy information for Experiment 2.

Target (ex. XANH, ‘blue’)	RT (*SD*)	E (in %)	Effect
C-overlap (e.g., XỈM)	546 (108)	0.8	33
C-control (e.g., PỈM)	579 (121)	0.9	
CV-Overlap (e.g., XÁU)	504 (91)	0.4	49
CV-Control (e.g., PÁU)	553 (111)	0.8	
Full-Overlap (e.g., XÀNH)	484 (79)	0.5	52
Full-Control (e.g., QÀNH)	536 (102)	0.8	

*SD*: standard deviation, RT: reaction times, E: error.

The factor Congruency was deviation-contrast-coded (-.5, .5). The optimal model was (invRT ~ Congruency * OverlapSize + [1|Participant] + [1|Item]). See Table 4 for more information.

**Table 4.** Coefficients of the main effects of the final model, together with the standard error, t-values and p-values (Experiment 2).

	Estimate	*SE*	*t*-value	*p*-value
Intercept	−1.84706	0.03342	−55.276	<.001
Congruency (Overlap)	−0.10202	0.02489	−4.098	<.001
OverlapSize (CV)	−0.11272	0.01758	−6.413	<.001
OverlapSize (FULL)	−0.17407	0.01756	−9.914	<.001
Congruency: OverlapSize (CV)	−0.07063	0.03515	−2.009	<.05
Congruency: OverlapSize (FULL)	−0.09180	0.03512	−2.614	<.01

*SE*: standard error.

Pairwise comparisons showed that for each of the overlap sizes (C, CV and Full) the congruency effect was present, C: *t*(48) = -3.75; *p* < .001; CV: *t*(48) = -6.08, *p* < .001; Full *t*(48) = -11.3; *p* < .001 with the C condition eliciting less facilitation than the CV and Full conditions (which were comparable with each other). These results indicate that, like Experiment 1, Vietnamese language production seems to use the phoneme as the fundamental phonological unit during the prosodification process (Levelt et al., 1999). The interaction indicates that this effect increases the more phonological information overlaps (C < CV = FULL).

Although it was pointed out earlier that Verdonschot and Kinoshita (2018) advocated the phonological Stroop task as a viable alternative for masked priming as there was “no intention to read the word” others have questioned this task’s suitability (Yoshihara et al., 2021). Particularly, in Verdonschot & Kinoshita’s work the script was moraic (katakana) or morphemic (kanji) and never *alphabetic* like in the current experiment (see onset effects for Japanese written in alphabetic romaji in Yoshihara et al., 2021). In addition, although no participant mentioned noticing this, it is still possible that our colour names may have been *cued by the onset* (and/or the following segments) of the visible nonwords enabling faster retrieval of the congruent colour names. This might have become evident to participants as most of the unrelated distractor onsets were not one of the five potential colour response options (except for DẶNG for the colour ĐEN/black). We therefore opted to run two additional picture-word interference experiments (i.e., thereby greatly increasing the response options averting a potential cueing problem), one (Experiment 3) with written distractors and one (Experiment 4) with auditory distractors.

## Experiment 3—Vietnamese PWI task using written distractors

### Method

#### Participants

Twenty-four participants (Age: 19.2 ± 1.5; 14 females, 10 males) either studying at Ho Chi Minh City Open University, Saigon University, The University of Medicine and Pharmacy at Ho Chi Minh City, or Industrial University of Ho Chi Minh City participated in this experiment for monetary compensation. None of these participants had taken part in Experiments 1 and 2. All had normal or corrected-to-normal vision. Participants took the LexTale test (Lemhöfer & Broersma, 2012; range: 0–100) and scored on average: 46 ± 7.1 indicating very low proficiency in English. Also, their self-assessment values (range: 0–100; *reading*: 46 ± 8.1; *writing*: 43 ± 7.7; *listening*: 43 ± 6.7, *speaking*: 35 ± 12.5) reflected low-proficiency and experience in using English as an L2. No abilities in other languages were reported.

#### Materials and design

Forty target words were selected (e.g., CUA “crab”) with four non-word conditions, C-Overlap (e.g., CÓT), C-Control (e.g., VÓT), Full-Overlap (e.g., CỤA), Full-Control (e.g., VỤA). C-Control and Full-Control always had the same rime and tone as their respective overlap condition. For each target word a black line-drawing (400 x 400 pixels) was selected from either the Snodgrass and Vanderwart (1980) dataset or if not present in this set created by the authors themselves.

#### Apparatus and procedure

E-Prime 3.0 was used to administer the experiment. First, a fixation (+) was shown for 1,000 ms. After that, the target (a picture) with distractor on it (SOA = 0) appeared and disappeared when it was named (or after maximally 2,500 ms). After this the experimenter judged whether the trial was correctly named or whether a voicekey error (e.g., couching, mis-triggering) or genuine error (e.g., wrong word) had occurred before the next trial started.

#### Results

About 2.8% of the RTs were discarded due to (1) a failure to respond, (2) stuttering or repairing a response, (3) triggering the voice key using a non-verbal response (e.g., coughing), and(4) or a failure to trigger the voice key. In addition, there were very few errors (1.0%; e.g., naming a wrong picture name) across the board which were equally distributed among conditions and therefore not analysed.

The treatment of correct RT data for this analysis was as follows. First, we excluded 5 trials (0.1%), which were faster than 300 or slower than 1,500 ms. This left 3687 data points for further analysis, see Table 5 for RTs and accuracy information. A comparison of raw RTs, log-transformed RTs, and inverse-transformed RTs (i.e., -1,000/RT) revealed that inverse-transformed RTs were closest to normality and were therefore used in subsequent analyses.

**Table 5.** RTs and accuracy information for Experiment 3 (picture naming with visual distractors).

Target (ex. CUA, ‘crab’)	RT (*SD*)	E (in %)	Effect
C-overlap (e.g., CÓT)	664 (141)	1.3	16
C-control (e.g., VÓT)	680 (146)	1.4	
Full-Overlap (e.g., CỤA)	567 (98)	0.6	72
Full-Control (e.g., VỤA)	639 (139)	0.7	

*SD*: standard deviation, RT: reaction times, E: error.

We considered the following predictors: “Trial” which denoted how far a participant had progressed in the experiment, “Congruency” with two levels (i.e., phonological overlap, control) and “OverlapSize” with two levels (i.e., Onset and Full). The factor Congruency was deviation-contrast-coded (-.5, .5). The final model was (invRT ~ Congruency * OverlapSize + [1|Participant] + [1|Item]). See Table 6 for more information.

**Table 6.** Coefficients of the main effects of the final model, together with the standard error, t-values and p-values (Experiment 3).

	Estimate	*SE*	*t*-value	*p*-value
Intercept	−1.52397	0.03097	−49.21	<.001
Trial	−0.07179	0.00369	−19.42	<.001
Congruency (Overlap vs. Control)	−0.03583	0.01053	−3.402	<.001
OverlapSize (C vs. FULL)	−0.104519	0.010500	−9.955	<.001
Congruency: OverlapSize (FULL)	−0.147736	0.014778	−9.997	<.01

*SE*: standard error.

Pairwise comparisons showed that for each of the overlap sizes (C and Full) the congruency effect was present, C: *t*(1,750) = -3.60; *p* < .001; Full *t*(1,807) = -17.97; *p* < .001 with the C condition (though significantly different from control) eliciting much less facilitation (16 ms) than the Full overlap condition (72 ms), *z* = -9.96, *p* < .001.

## Experiment 4—Vietnamese PWI task using auditory distractors

### Method

#### Participants

Twenty-four participants (Age: 19.4 ± 1.5; 14 females 10 male) either studying at Ho Chi Minh City Open University, Saigon University, The University of Medicine and Pharmacy at Ho Chi Minh City, or Industrial University of Ho Chi Minh City participated in this experiment for monetary compensation. None of these participants had taken part in any of the previous experiments. All had normal or corrected-to-normal vision. Participants took the LexTale test (Lemhöfer & Broersma, 2012; range: 0–100) and scored on average: 52 ± 7.0 indicating very low proficiency in English. Also, their self-assessment values (range: 0–100; *reading*: 53 ± 11.9; *writing*: 47 ± 14.6; *listening*: 40 ± 18.5, speaking: 53 ± 11.9) reflected similar low-proficiency and experience in using English as an L2. No abilities in other languages were reported.

#### Materials and design

Identical to Experiment 3. However, all non-word distractors were now *recorded sound files* instead of written words. Care was taken to create auditory distractors of approximately equal duration (~486 ms on average). Auditory distractors coincided with the picture target (SOA = 0).

#### Apparatus and procedure

E-Prime 3.0 was used to administer the experiment. First, a fixation (+) was shown for 1000 ms. After that, the target (a picture) appeared and disappeared when it was named (or after maximally 2500 ms). After this the experimenter judged whether the trial was correctly named or whether a voicekey error (e.g., couching, mistriggering) or genuine error (e.g., wrong word) had occurred before the next trial started.

#### Results

About 2.3% of the RTs were discarded due to (1) a failure to respond, (2) stuttering or repairing a response, (3) triggering the voice key using a non-verbal response (e.g., coughing), and (4) or a failure to trigger the voice key. In addition, there were reasonably few errors (3.3%; e.g., naming a wrong picture name) and therefore not analysed.

The treatment of correct RT data for this analysis was as follows. First, we excluded 20 trials (0.5%), which were faster than 300 or slower than 1,500 ms. This left 3605 data points for further analysis, see Table 7 for RTs and accuracy information. A comparison of raw RTs, log-transformed RTs and inverse-transformed RTs (i.e., -1,000/RT) revealed that inverse-transformed RTs were closest to normality and were therefore used in subsequent analyses.

**Table 7.** RTs and accuracy information for Experiment 4 (picture naming with auditory distractors).

Target (ex. CUA, ‘crab’)	RT (*SD*)	E (in %)	Effect
C-overlap (e.g., CÓT)	684 (146)	1.3	8
C-control (e.g., VÓT)	692 (138)	1.4	
Full-Overlap (e.g., CỤA)	638 (110)	0.6	50
Full-Control (e.g., VỤA)	688 (133)	0.7	

*SD*: standard deviation.

The final model was (invRT ~ Congruency * Overlap + [1|Participant] + [1|Item]). Any random slopes entered in the participant or item random effect structure resulted in statistical models which either did not converge or were not significantly better. See Table 8 for more information.

**Table 8.** Coefficients of the main effects of the final model, together with the standard error, t-values and *p*-values (Experiment 4).

	Estimate	*SE*	*t*-value	*p*-value
Intercept	−1.5425723	0.0277469	−55.594	<.001
Trial	0.0020525	0.0003183	6.449	<.001
Congruency (Overlap vs. Control)	−0.0675846	0.0073300	−9.220	<.001
OverlapSize (C vs. FULL)	−0.0050432	0.0104391	−0.483	.629
Congruency: OverlapSize (FULL)	−0.0860832	0.0146613	−5.871	<.001

*SE*: standard error.

Pairwise comparisons showed that for each of the overlap sizes (C and Full) the congruency effect was present, C: *t*(1,704) = -2.2; *p* < .05; Full: *t*(1,775) = -10.96, *p* < .001; with the C condition eliciting less facilitation than the Full conditions. These results indicate that, although the effect is rather small for the C-Overlap condition (8 ms), like the other experiments, Vietnamese language production uses the phoneme as the initial phonological unit during the prosodification process (Levelt et al., 1999).

## General discussion

This study set out to investigate the fundamental phonological unit of language production in Vietnamese, a language which has similar properties to Chinese such as a syllable-timing, the absence of re-syllabification and inflection; but uses an alphabetic script which is written using breaks between syllables (note: even syllables in compound words are separated by a space). There were several clear indications to investigate whether the syllable would be at the base of the phonological encoding process in this language. If so, then that would have constituted a unique combination of a language having a syllabic fundamental phonological unit though written using an alphabetic script. To study phonological encoding in Vietnamese, we administered four experimental paradigms: (1) a masked priming word naming experiment, (2) a phonological Stroop task, (3) a PWI experiment with written distractors, and (4) a PWI experiment with auditory distractors. Experiment 1 obtained significant onset priming (e.g., target: HOA “flower” with prime: heo “pig” vs. súng “gun”) as well as significant CV priming (hộp “box”) and full overlap priming (e.g., hoả “fire”). In addition, the second Experiment (phonological Stroop task) also obtained facilitation of naming latencies when the first segment between colour and non-word were overlapping (e.g., xỉm for the colour xanh “blue”) and additional benefits (i.e., more facilitation) was obtained when more segments were overlapping (e.g., xáu and xành). Both PWI experiments obtained facilitatory onset as well as syllable effects (although the size was rather small for the auditory onset effect).

One result pattern which differed between Experiment 1 (masked priming) and Experiment 2 (phonological Stroop) is that the effect sizes were similar between the conditions (C, CV, Full) in Experiment 1, but they were different for Experiment 2 with C showing less facilitation than CV and Full (which were similar). As stated in the discussion of Experiment 1, ordinarily identity conditions show greater priming effects than, for example, onset conditions. However, in Experiment 1 the full condition was *not* an identity condition as the (orthographically marked) tone and therefore the meaning was different (e.g., target: MỰC “ink” and prime: mức “level,” the tone is clearly differentially marked: ự [i.e., low dropping pitch marked by the dot below the letter] / ứ [i.e., high rising pitch marked by the acute accent]).

Also, in our experiments a distinction can be made between tasks that involve reading aloud words (Experiment 1) versus the naming of objects (i.e., colours, pictures; Experiments 2–4). In other words, whether the to-be-named target is written or presented as a picture or a colour. Production of a picture name or colour name is typically not assembled in a piece-by-piece fashion as in when a letter/character string is read aloud. For Experiment 1 (masked priming) the fact that the benefit due to CV-overlap (and even the CVC overlap) was statistically not greater than the benefit due to C-overlap might indicate that this task was more involved with reading aloud a written word and/or the prime may not have been fully processed. There are several reports which show that additional overlap between prime and target do not necessarily evoke larger priming (e.g., Kinoshita, 2000; Nakayama et al., 2016).

One possible reason why there was a distinction between the C and the CV/Full conditions in Experiment 2 might be that RTs for the second experiment were about 100 ms longer (Experiment 1: 431 ± 58; Experiment 2: 533 ± 102) and had a larger variability in general compared to the first experiment. However, as pointed out in the discussion of Experiment 2 it is also possible that our colour names might have been *cued by the onset* (and the following segments) of the visible nonwords enabling faster retrieval of the congruent colour names.

Experiments 3 and 4 were PWI experiments and greatly increased the response options averting the potential cueing problem of Experiment 2 (especially Experiments 4 which used auditory distractors). Both PWI experiments showed significant onset effects and full-overlap effects although the latter were much more pronounced than the onset effects (compared to the results of Experiments 1 and 2).

Overall, the general pattern from these results is that even though Vietnamese on the surface seems to have many surface characteristics of Chinese (e.g., tonal language, syllabic-timing, no re-syllabification, no inflections), at the core of how phonology is constructed it behaves similarly to Germanic languages (e.g., English, Dutch, German). That means that the phoneme is likely to be the initial phonological unit selected in the prosodification process.

This is an interesting finding as many of the arguments laid out in previous papers as to why Chinese does not have onset priming (e.g., Chen et al., 2002; O’Seaghdha et al., 2010; You et al., 2012) also hold for Vietnamese. In Vietnamese word form construction depends primarily on syllabic units, which are simple in structure (like Chinese). Syllables in Vietnamese are typically CV(C), in which specification of tone is required (the onset, as well as the coda consonant, however, are optional) and the language does not have re-syllabification, nor does it have inflections.

One parsimonious explanation for our data is that, despite any (apparent) similarities to Chinese, the basic phonological unit to be used in the prosodification process is simply the phoneme. Though Sidwell (2008) pointed out that many aspects of the morphological system of Vietnamese seems to be like Chinese, this does not guarantee it must behave in a similar way with respect to phonological encoding. For example, one important argument for the presence of onset effects during phonological encoding may be that Vietnamese tones are distinct from Chinese tones in that they do not solely rely on pitch contour. In other words, some Vietnamese tones are characterised by attributes other than pitch; therefore, in Vietnamese the tonal frame might entail more than simply assigning tonal pitch contour to the syllable (A. H. Pham, 2001; A. H. Pham & Horn, 2004). For example, the Vietnamese sắc tone starts in the middle and subsequently rises. The ngã tone also starts in the middle (though typically higher than the sắc tone for some dialects) and rises but for many speakers induces a glottal stop (/ʔ/) in the middle of the vowel. A. H. Pham (2001) clearly illustrates this by showing a discontinuous portion in the tone’s pitch curve (Figure 7a; p 94). Another example is the huyền tone which starts low-mid and falls subsequently. This tone is akin to the nặng tone which follows the same pattern, but it swiftly falls in pitch. However, the latter (nặng) tone has a short “creaky” vowel, as opposed to a long “breathy” vowel in the first (huyền) tone. Vietnamese is also sometimes called a pitch register language in which different features combine and (e.g., vowel length and quality, tone, glottalization) all have an interplay with each other (A. H. Pham, 2001). This might make Vietnamese tone-to-syllable assignment considerably more intricate than it is for Chinese (see Roelofs, 2015) and consequently segments might need to be initially available in Vietnamese to be able to assign the correct combination of features to the to be uttered word. For example, A. H. Pham (2001) argues that the critical portion in a syllable for distinguishing Vietnamese tones is different depending on the tone (i.e., the middle point in a word in hỏi and ngã, but the endpoint for some other tones like sắc).

**Figure 1.**
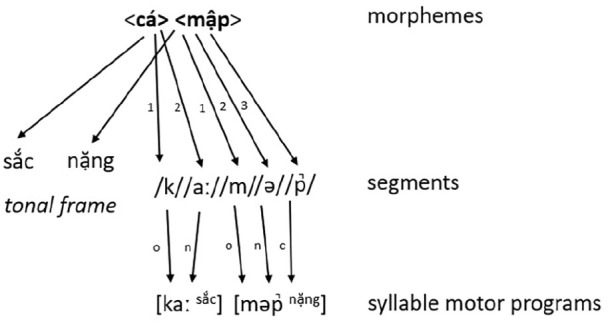
Illustration (following Roelofs, 2015) of the Vietnamese (compound) word cá mập “shark” during the prosodification stage of the Levelt et al. (1999) model (o = onset, *n* = nucleus, c = coda). The words sắc and nặng above the tonal frame indicate the Vietnamese tone names, the numbers 1–3 indicate the position of the segments in the respective morphemes.

Here, we outline a tentative proposal for Vietnamese phonological encoding in Figure 1 (using a similar schematic as used in Roelofs, 2015).

We assume the existence of lexemes, tonal frames, segments, and syllable motor programmes in the Vietnamese form network. The tones in the model are denoted by their names (i.e., ngang, huyền, sắc, nặng, hỏi, and ngã). During form encoding tonal frame nodes as well as their associated segments are selected, and the segments will be incrementally combined with the tonal nodes. We propose the initial involvement (i.e., proximate unit; O’Seaghdha et al., 2010) of *segments*, and *not* atonal syllables as in Chinese, due to the persistent onset effects across all our experiments which could be due to the intricate assignment of tone (for which the argument has been laid out in the previous paragraph). Ultimately, phonological encoding will result in a phonological word representation, which has specified the segments grouped into syllables using the appropriate pattern for the associated tone. The example in Figure 1 denotes a di-syllabic (the most common word structure in Vietnamese) compound word *cá mập* (“shark” in Vietnamese). This word consists of two separate morphemes (i.e., *cá* means fish, and *mập* means fat) which in turn activate their associated tone as well as their individual constituents (denoted with numbers) and will be prosodified into [kaː ^sắc^] [məp̚ ^nặng^].

However, another possibility which may account for our results, already mentioned in the introduction, is that even if the language portrays many similar syllabic characteristics to Chinese, it could be that by using the modern Vietnamese alphabetic script (i.e., chữ quốc ngữ) imposed by the French in the early 1900’s, Vietnamese people may have developed a deep grained awareness of phonemes. The participants in our study were all young university students who did not have any mastery of Chinese script and grew up with an alphabetic script to read and write their language. So, it might be the case that the grain size of the script used to write Vietnamese has shaped Vietnamese phonological encoding. There is evidence from Japanese (Inagaki et al., 2000) showing that pre-school Japanese children did not necessarily show mora-related segmentation for aurally presented words. That is, about half of the children segmented words into syllables. However, after they learned Kana characters almost all of them showed a mora-based segmentation, indicating a change in awareness due to acquiring literacy. One example to the contrary, although somewhat anecdotal, is the observation that *nói lái* (Vietnamese Spoonerisms) such as bí mật “secret” (noun) > bật mí “to reveal a secret” (verb) is already a very old phenomenon, which has been recorded even before the alphabetic script was introduced into Vietnam. As Macken and Nguyễn (2006, p. 54) point out:

the fact that nói lái was used and valued highly for centuries by Vietnamese speakers who could not read means that the phonological units of nói lái are accessed and used independently of the writing system and independently of learning to read a written language.

In any event, this possibility needs to be further investigated in future endeavours.

To conclude, as far as we know, this study is the first study to report chronometric data (i.e., RTs) on Vietnamese phonological encoding. Our data show that prosodification in Vietnamese proceeds similarly to Germanic languages (like Dutch, English, and German). In other words, the phoneme is the fundamental phonological unit in this language (O’Seaghdha et al., 2010; Roelofs, 2015), despite Vietnamese having many apparent resemblances to Chinese. We believe that this is a genuine phenomenon of the phonological encoding system itself, possibly due to the unique assignment of tone in Vietnamese, though we cannot rule out that the alphabetic grain size of the chữ quốc ngữ script used to write the language has not shaped the phonological unit used Vietnamese language users.

## Supplemental Material

sj-docx-1-qjp-10.1177_17470218211053244 – Supplemental material for Phonological encoding in Vietnamese: An experimental investigation Click here for additional data file. Supplemental material, sj-docx-1-qjp-10.1177_17470218211053244 for Phonological encoding in Vietnamese: An experimental investigation by Rinus G Verdonschot, Hoàng Thị Lan Phương and Katsuo Tamaoka in Quarterly Journal of Experimental Psychology

## Notes

1. All data and analysis methods are available from first author upon request.
