# Supplementary material for: Phonological encoding in Vietnamese: An experimental investigation
Source: Q J Exp Psychol (Hove). 2021 Oct 21;75(7):1355–66. doi: 10.1177/17470218211053244 (PMC9131412; doi:10.1177/17470218211053244)
Supplement: sj-docx-1-qjp-10.1177_17470218211053244 – Supplemental material for Phonological encoding in Vietnamese: An experimental investigation [file sj-docx-1-qjp-10.1177_17470218211053244.docx]

Supplementary Material for:

1. **Phonological encoding in Vietnamese: an experimental investigation.**
2. Rinus G. Verdonschot
3. Max Planck Institute for Psycholinguistics. Nijmegen. The Netherlands
4. Hoàng Thị Lan Phương & Katsuo Tamaoka
5. Graduate School of Humanities, Nagoya University, Nagoya, Japan

Supplementary Material A – Stimuli used in Experiment 1

| Target | C-Overlap | C-Control | CV-Overlap | CV-Control | Full-Overlap | Full-Control |
| --- | --- | --- | --- | --- | --- | --- |
| CUA (crab) | cam (orange) | sẹo (scar) | cúc (button) | son (lip stick) | cưa (saw) | sói (wolf) |
| SỎI (gravel) | sẹo (scar) | cam (orange) | son (lip stick) | cúc (button) | sói (wolf) | cưa (saw) |
| BẠN (friend) | beo (leopard) | nấm (mushroom) | bát (bowl) | nốt (note) | bàn (table) | nôi (baby crib) |
| NỒI (pot) | nấm (mushroom) | beo (leopard) | nốt (note) | bát (bowl) | nôi (baby crib) | bàn (table) |
| KIM (needle) | kẹo (candy) | đực (male) | kính (glasses) | đậu (bean) | kìm (pincers) | đao (sword) |
| ĐÀO (peach) | đực (male) | kẹo (candy) | đậu (bean) | kính (glasses) | đao (sword) | kìm (pincers) |
| HOA (flower) | heo (pig) | súng (gun) | hộp (box) | sân (ground) | hoả (fire) | sấm (thunder) |
| SÂM (ginseng) | súng (gun) | heo (pig) | sân (ground) | hộp (box) | sấm (thunder) | hoả (fire) |
| LAN (orchid) | lốp (tire) | bướm (butterfly) | lạc (peanut) | bông (cotton) | lân (unicorn) | bốt (boots) |
| BỘT (flour) | bướm (butterfly) | lốp (tire) | bông (cotton) | lạc (peanut) | bốt (boots) | lân (unicorn) |
| RỐI (puppet) | rắn (snake) | bình (vase) | rồng (dragon) | bắp (corn) | roi (rod) | báo (newspaper) |
| BAO (bag) | bình (vase) | rắn (snake) | bắp (corn) | rồng (dragon) | báo (newspaper) | roi (rod) |
| TOÀ (courtyard) | tất (socks) | muối (salt) | tóc (hair) | mây (cloud) | toa (carriage) | mấn (hat) |
| MẬN (plum) | muối (salt) | tất (socks) | mây (cloud) | tóc (hair) | mấn (hat) | toa (carriage) |
| MAI (apricot) | mọt (worm) | sợi (fibre) | mắt (eye) | sếp (boss) | mái (roof) | sen (lotus) |
| SÊN (slug) | sợi (fibre) | mọt (worm) | sếp (boss) | mắt (eye) | sen (lotus) | mái (roof) |
| TAI (ear) | tép (tiny shrimp) | cân (scale) | táo (apple) | con (children) | tải (transport) | cớm (police) |
| CƠM (rice) | cân (scale) | tép (tiny shrimp) | con (children) | táo (apple) | cớm (police) | tải (rod) |
| VỊT (duck) | võng (hammock) | tấm (sheet) | viện (hospital) | tàu (train) | vít (screws) | tảo (seaweed) |
| TÁO (apple) | tấm (sheet) | võng (hammock) | tàu (train) | viện (hospital) | tảo (seaweed) | vít (screws) |
| ĐỒI (hill) | đèn (lamp) | mèo (cat) | đồn (stronghold) | mứt (jam) | đội (team) | mức (level) |
| MỰC (ink) | mèo (cat) | đèn (lamp) | mứt (jam) | đồn (stronghold) | mức (level) | đội (team) |
| CẦU (bridge) | cóc (toad) | môi (lips) | cây (tree) | mục (catalogue) | câu (sentence) | mụn (acnes) |
| MÙN (sawdust) | môi (lips) | cóc (toad) | mục (catalogue) | cây (tree) | mụn (acnes) | câu (sentence) |

Supplementary Material B – Stimuli Experiment 2

| **Color** | **Vietnamese** | **C-overlap** | **C-control** | **CV-overlap** | **CV-control** | **Full-overlap** | **Full-control** |
| --- | --- | --- | --- | --- | --- | --- | --- |
| PINK | HỒNG | HẾP | GẾP | HỖT | GỖT | HỐNG | PỐNG |
| PINK | HỒNG | HỊC | GỊC | HỖM | GỖM | HỖNG | PỖNG |
| PINK | HỒNG | HÃT | GÃT | HÔU | GÔU | HỘNG | PỘNG |
| PINK | HỒNG | HƯM | GƯM | HỖC | GỖC | HỔNG | PỒNG |
| PINK | HỒNG | HÂC | GÂC | HÔP | GÔP | HÔNG | PÔNG |
| PURPLE | TÍM | TON | MON | TIC | LIC | TIM | RIM |
| PURPLE | TÍM | TÙC | MÙC | TỈP | LỈP | TÌM | RÌM |
| PURPLE | TÍM | TỚP | MỚP | TÌN | LÌN | TỈM | RỈM |
| PURPLE | TÍM | TẺC | MẺC | TĨR | LĨR | TĨM | RĨM |
| PURPLE | TÍM | TAT | MAT | TỊN | LỊN | TỊM | RỊM |
| BLACK | ĐEN | ĐỈP | NỈP | ĐÈC | NÈC | ĐÉN | CÉN |
| BLACK | ĐEN | ĐẺT | NẺT | ĐÈP | NÈP | ĐÈN | CÈN |
| BLACK | ĐEN | ĐỦM | NỦM | ĐẺM | NẺM | ĐẺN | CẺN |
| BLACK | ĐEN | ĐÀC | NÀC | ĐẼT | NẼT | ĐẼN | CẼN |
| BLACK | ĐEN | ĐÓP | NÓP | ĐẸC | NẸC | ĐẸN | CẸN |
| BLUE | XANH | XỈM | PỈM | XÁU | PÁU | XÀNH | QÀNH |
| BLUE | XANH | XÈC | PÈC | XÀP | PÀP | XẢNH | QẢNH |
| BLUE | XANH | XỬP | PỬP | XÃM | PÃM | XÃNH | QÃNH |
| BLUE | XANH | XỒT | PỒT | XẠC | PẠC | XẠNH | QẠNH |
| BLUE | XANH | XỰM | PỰM | XẢM | PẢM | XÁNH | QÁNH |
| WHITE | TRẮNG | TRÒM | BÒM | TRẲT | BẲT | TRĂNG | DĂNG |
| WHITE | TRẮNG | TRỦC | BỦC | TRẮM | BẮM | TRẶNG | DẶNG |
| WHITE | TRẮNG | TRẸP | BẸP | TRẴC | BẴC | TRẰNG | DẰNG |
| WHITE | TRẮNG | TRỜT | BỜT | TRẶN | BẶN | TRẲNG | DẲNG |
| WHITE | TRẮNG | TRỮP | BỮP | TRĂC | BĂC | TRẴNG | DẴNG |

Supplementary Material C **–** Stimuli experiments 3 and 4

| Target | Meaning | C-overlap | C-control | Full-overlap | Full-control |
| --- | --- | --- | --- | --- | --- |
| CUA | crab | CÓT | VÓT | CỤA | VỤA |
| LƯỢC | comb | LIẾT | HIẾT | LƯỚC | HƯỚC |
| NẤM | mushroom | NẸC | LẸC | NẪM | LẪM |
| VOI | elephant | VÉP | BÉP | VỌI | BỌI |
| KEM | ice cream | KỊT | RỊT | KẸM | RẸM |
| HOA | flower | HẾP | RẾP | HOÃ | ROÃ |
| BƯỚM | butterfly | BIỆC | XIỆC | BƯỜM | XƯỜM |
| TIM | heart | TỘP | LỘP | TỈM | LỈM |
| DÂU | strawberry | DỈM | RỈM | DẨU | RẨU |
| SEN | lotus | SỰT | MỰT | SẸN | MẸN |
| BÀN | table | BỦM | CỦM | BÃN | CÃN |
| SAO | star | SÙN | RÙN | SÃO | RÃO |
| CÂY | tree | CỌT | DỌT | CẨY | DẨY |
| LỬA | fire | LỆN | HỆN | LƯA | HƯA |
| CỬA | door | CÔM | ĐÔM | CỪA | ĐỪA |
| HEO | pig | HỤN | BỤN | HẸO | BẸO |
| NÚI | mountain | NỆP | XỆP | NỦI | XỦI |
| ĐĨA | plate | ĐỤM | VỤM | ĐÌA | VÌA |
| NÓN | hat | NỆU | SỆU | NÒN | SÒN |
| BÓNG | ball | BẾCH | NẾCH | BÕNG | NÕNG |
| MŨI | nose | MẾT | SẾT | MỤI | SỤI |
| VÁY | dress | VỤC | HỤC | VÀY | HÀY |
| MÂY | cloud | MẸC | HẸC | MẬY | HẬY |
| HỘP | box | HIN | DIN | HỐP | DỘP |
| DAO | knife | DÚC | GÚC | DẢO | GẢO |
| ĐÈN | lamp | ĐỐP | GỐP | ĐÉN | GÉN |
| CAM | orange | CỦN | VỦN | CÃM | VÃM |
| BẮP | corn | BỰT | NỰT | BẶP | NẶP |
| RÙA | turtle | RÉP | ĐÉP | RÚA | ĐÚA |
| ĐÀN | guitar | ĐỰP | GỰP | ĐÃN | GÃN |
| GẤU | bear | GON | XON | GẬU | XẬU |
| CÂN | scale | CỰP | TỰP | CẪN | TẪN |
| TÔM | shrimp | TẸC | DẸC | TỐM | DỐM |
| ĐẬU | pea | ĐÉM | HÉM | ĐẪU | HẪU |
| LON | can | LỊP | MỊP | LỎN | MỎN |
| SÂU | caterpillar | SÈN | VÈN | SẬU | VẬU |
| TÀU | ship | TẺN | SẺN | TẠU | SẠU |
| TAY | hand | TỰM | DỰM | TÁY | DÁY |
| TEM | stamp | TỤA | XỤA | TÈM | XÈM |
| SỨA | jelly-fish | SỘT | NỘT | SỪA | NỪA |
